# Transnational Networks’ Contribution to Health Policy Diffusion: A Mixed Method Study of the Performance-Based Financing Community of Practice in Africa

**DOI:** 10.34172/ijhpm.2020.57

**Published:** 2020-04-27

**Authors:** Lara Gautier, Manuela De Allegri, Valéry Ridde

**Affiliations:** ^1^Department of Social and Preventive Medicine, University of Montreal, Montreal, QC, Canada.; ^2^CESSMA (IRD-Paris-Diderot University), Université de Paris, Paris, France.; ^3^Heidelberg Institute of Global Health, Medical Faculty and University Hospital, Heidelberg University, Heidelberg, Germany.; ^4^CEPED (IRD-Université de Paris), Université de Paris, ERL INSERM SAGESUD, Paris, France.

**Keywords:** Transnational Policy Networks, Communities of Practice, Social Network Analysis, Semantic Analysis, Performance-Based Financing, Sub-Saharan Africa

## Abstract

**Background:** Transnational networks such as Communities of Practice (CoPs) are flourishing, yet their role in diffusing health systems reforms has been seldom investigated. Over the past decade, performance-based financing (PBF) has rapidly spread in Africa. This study explores how, through the PBF Community of Practice’s attributes, structure, and strategies, PBF diffusion was fostered in sub-Saharan Africa (SSA).

**Methods:** Informed by the diffusion entrepreneurs’ (DEs) framework dimensions, we used a mixed methods convergent design to investigate how the attributes, structure, and strategies of this community fostered the diffusion of PBF. The quantitative strand of work included firstly a semantic discourse analysis of textual data extracted from CoP’s online discussion forum (n=1346 posts). Secondly, the relational data extracted from these 1346 forum posts was examined using social network analysis (SNA). We confronted these quantitative results with a thematic analysis of qualitative interviews (n=40) and data extracted from the CoP’s key documentation (n=17).

**Results:** CoP members’ attributes included: representation systems anchored in clinical and economic sciences, strong expectations that the CoP would boost professional visibility and career, and significant health systems knowledge and social resources. The CoP’s core group, dominated by high-income country (HIC) members, critically matched PBF principles to major health systems issues in Africa. The broad consensus in online PBF thematic discussions created a strong sense of community, a breeding ground for emulation among CoP members. The CoP also sought to produce and promote experiential knowledge exchanges about PBF amongst African practitioners. Findings from network analyses showed that the promoted Africa-driven community was led by HIC members, although their prominence tended to decrease with time.

**Conclusion:** This empirical research highlighted some of the constituting features, structure, and strategies of policy networks in influencing health policy diffusion. Despite good intentions to disrupt the established governance landscape, influential actors coming from HICs continued to drive the framing, and shaped health systems policy experimentation, emulation, and learning in African countries. Beyond mere knowledge exchange platforms, CoP can act as meaningful transnational policy networks pursuing the diffusion of health systems reforms, such as PBF.

## Background

Global health governance is characterised by polycentrism. Polycentric governance refers to a policy landscape whereby autonomous governing units spread their normative and regulatory power at different scales.^1^ This feature enables transnational networks to gain influence in global health policy-making.^2^ However, the role of networks in diffusing health systems reforms has been seldom investigated.^3^ A recent study on global networks has concluded that “transnational connectivity can be expected to affect health policies.”^4^ Communities of Practice (CoPs) are “groups of people who share a concern or a passion for something they do and learn how to do it better as they interact regularly.”^5^ They represent an important global health platform because of their potential to mobilise people’s knowledge from multiple sectors in view of supporting health systems reforms’ implementation.^6^ CoPs are interactive and inclusive networks: they organise and online discussion fora for health practitioners, policy-makers, researchers, and multilateral agencies.^7^

CoPs have flourished in the past 15 years; several of them have strived to develop a “repertoire of resources” for health systems financing reforms. While the CoPs’ primary aim remains that of fostering knowledge exchange, some CoPs (like many other transnational actors) have also served to promote the diffusion of health systems reforms in sub-Saharan Africa (SSA).^8,9^ As they engage in “regular communication and frequent exchange of information,” thus leading to “the establishment of stable relationships […] and to the coordination of their mutual interests”^10^ CoPs have the potential to become policy networks aiming at spreading policy ideas in multiple locations.^11^ Such phenomenon is referred to as policy diffusion.^12^ There is a limited body of literature on health policy diffusion in low- and middle-income countries (LMICs).^13^ In particular, the complexity of transnational networks’ influence in diffusion processes has received little attention from global health scholars.^14^ Building on 2 main strands of public policy literature, policy diffusion^12^ and *sociologie de l’action publique*,^15^ the framework developed by Gautier et al helps to unravel the multiple features of those influential actors who strive to foster policy diffusion in LMICs, aka “diffusion entrepreneurs” (DEs).^16^ This theoretically-driven framework features the diverse and interrelated dimensions of the political economy of performance-based financing (PBF) diffusion in SSA.^17^ It has been successfully used in analyses of PBF diffusion patterns at the global and national levels.^8,18^ DEs act in polycentric contexts: they may operate as autonomous units of political authority. Fuelling their power into multiple units of governance,^11^ transnational policy networks are salient illustrations of the DE concept. Empirical applications to the study of this type of networks, using the case of a CoP which has a strong transnational activity in SSA, are timely.

PBF has spread in SSA very rapidly.^16^ PBF is a health financing reform suggesting a shift from the traditional input-based transfer of financial resources for service provision to an output-based approach conditional on providers’ performance. PBF diffusion has been fostered by a wide range of Des.^16^ Global DEs endeavoured to create fora to bring pilot PBF experimenters together.^8^ The PBF CoP emerged in 2010 as the practitioners’ alternative to existing knowledge exchange networks, including the World Bank-led Interagency Working Group on Results-Based Financing.^19^

This study was set to answer the following research question: how did the PBF CoP’s attributes, structure, and strategies contribute to foster the diffusion of PBF in the African continent? This question bears conceptual implications in relation to how we frame and analyse transnational networks (specifically, CoP), since we choose to treat them as *policy networks* developing explicit strategies to influence the spread of policies, rather than ‘apolitical’ knowledge exchange networks, as this is often the case. Like Stone, we conceive policy networks as “agents in the galaxy of transnational networks that are the vehicles for policy processes.”^11^

## Methods

Policy networks may be investigated by looking at their attributes, structure and agency.^10^ We unravel the CoP’s attributes by looking at its constituting features, and its structure and agency by looking at its interconnected strategies. We followed Adam and Kriesi’s guidance^10^ to study policy networks using mixed methods relying on a concurrent quantitative and qualitative analyses^20^ to unravel the CoP’s attributes (ie, constituting features), structure and strategies. All definitions of key concepts are provided in Table 1.

**Table 1:** Definitions of Key Concepts Used for Analysis^16,18,21^

**Items**	**Definition**
Networks’ attributes	Key characteristics of networks and their members (including representation systems and resources)
Networks’ structure	How relations between network members are combined or arranged, to reflect patterns of interaction
Agency	Capacity to act autonomously
Representation systems	Ideational foundations bearing underlying assumptions about the world, drawn from cultural backgrounds
Knowledge resources	Any form of knowledge (eg, scientific evidence, lay/practice evidence, etc)
Political resources	Capacity to mobilise key policy actors (eg, building upon previous collaboration with policy-makers)
Material resources	Human resources, material equipment and funding
Social resources	Social capital and actors’ ability to connect with other people
Temporal resources	Actors’ ability to find/make time

Note: For additional definitions of the framework components, please refer to Gautier et al.^16^

The DE framework^16^ offers conceptual categories to explore the constituting features and strategies used by DEs to diffuse policies. First, drawing on the seminal work by the French sociologist of *action publique* Hassenteufel,^15^ the framework outlines 4 major interrelated constituting features, namely representation systems, interests, resources, and authority. DEs share common representation systems that match the core ideas of the favoured policy, and common interests (which may be personal and/or societal) to spur diffusion.^16^ Thanks to available knowledge, political, material, social, and temporal, resources, DEs acquire authority in global policy-making arenas (Table 1).

Second, drawing on the literature on policy diffusion mechanisms,^12,22^ Gautier et al consider that these constituting features enable DEs to design strategies to frame the policy in attractive ways, induce policy emulation (eg, how socialisation sparks interest for a policy), and shape policy experimentation and learning (eg, sharing policy knowledge across different settings). These strategies are interconnected: they may feed into each other to further policy diffusion. For example, a common attractive framing of a given policy fosters emulation among actors, which in turn sparks enthusiasm to experiment the policy. Further details and illustrations of DEs’ constituting features and strategies are provided in Gautier et al.^16^ DEs are individuals (eg, consultants), organisations (eg, aid donors), and (trans)national networks. Indeed, transnational policy networks gain power from “their (semi)official position; their control of, and other organisational resources; their technical expertise or epistemic authority; or their often lengthy international experience as career officials and consultants.”^11^ In Gautier et al,^16^ authors suggest that the PBF CoP acts as DE.

First, to unpack one of the CoP’s main attributes (its representation systems) and its strategies to frame PBF, we performed a quantitative semantic discourse analysis^23^ of the topical content of the CoP’s online forum. The term “topical” was used to refer to content from discussion threads that related to PBF experience (ie, institutional processes, funding, implementation, or evaluation) or theories. Archives of the discussion forum from January 2010 until September 2016, in both English and French, were screened for topical content. 1346 messages (344 threads) were extracted. Data was coded using major semantic categories (see Supplementary files 1 and 2 for details) relating to the anchor disciplines of PBF (eg, economics, management, clinical, social), which emerged from prior analysis of PBF DEs’ discourse. Words and expressions pertaining to key semantic fields were listed *a priori*, and any sentence containing these words/expressions was automatically coded using the software QDA Miner^©^. We used the software’s coding retrieval and statistical features (on code frequencies, percentage coverage of coded words, etc), to produce the results.

Second, we performed a social network analysis (SNA) to investigate the CoP’s structure.^24^ We used SNA to analyse communication ties joining members to each other, as well as the ties joining members to non-members. These ties featured members’ citations (ie, mentions in forum messages) of other persons’ names (both members and non-members, PBF practitioners or others). Our goal was to visualise the CoP’s network structure in terms of policy emulation (eg, is it a cohesive community?) and learning (eg, who cites who, and what does it say about the type of knowledge being shared and the community’s openness?) We made the following analytical assumptions: citing a specific person (whether a CoP member or not) was conceived as a proxy for both policy emulation (ie, explicitly recognising that this person is part of the same policy community) and policy learning (ie, explicitly recognising that this person’s contribution expands PBF knowledge).

Based on the selected topical discussion threads, anonymous identifiers of forum contributors (n = 186) citing CoP members and non-members were extracted using Excel. This means that our analysis focuses on a CoP active group rather than the whole community: the SNA only features 287 members (out of more than 2000 members). Several CoP members commented. Their suggestion to split the data into 2 sections (from 2010 to 2012; and from 2013 to 2016) was applied so as to observe evolution patterns. This feature added to the credibility of our analysis and confirmed social acceptance of our results among the CoP. We converted relational data to an adjacency matrix and brought it into R. We obtained weighted and directed graphic representations to inform policy emulation and policy learning, respectively. For the weighted network representation, we hypothesised a strongly connected network. For the directed network representation, we hypothesised a high number of citations among CoP members. Table 2 provides the key definition for each SNA concept.

**Table 2:** Basic Concepts in SNA

**Items**	**Definition**
Relational data	Table featuring who cites who and how many times?
Adjacency matrix	For a network of N nodes, it represents a matrix of ones and zeros where a one indicates the presence of the corresponding edge in the network^25^
Weighted network representation	A graph featuring each of the nodes’ strength, ie, the sum of the values of the links in which a node is engaged with
Directed network representation	A graph that enables to visualise who cited whom and who got cited by whom

Abbreviation: SNA, social network analysis.

Third, we collected qualitative data: in-depth interviews (n = 40) and documents on the CoP (n = 17). Documents included: 14 key blogs on the PBF CoP, 2 meeting reports produced by the CoP, and one concept note on the CoP. In-depth interviews with informants (ie, PBF members of the CoP, as well as PBF experts knowledgeable about the CoP but non-members) were carried out from November 2016 to July 2017 in situ or by phone, in English or French. Interview guides included 37 open-ended questions informed by the framework dimensions (Supplementary file 3). Snowball sampling was the preferred strategy, given that CoP members and PBF experts frequently interacted with each other. Our respondents’ affiliations (Table 3) are representative of the background profile of CoP members, ie, practitioners working for the government or private entities, senior cadres, researchers, and technical assistants from international organisations.^26^

**Table 3:** Respondents’ Characteristics

**Current Affiliation (N = 40)**	**Main Educational Background (N = 40)**	**Years of Experience in International Development, All Excluding “NATGOV” Category (N = 35)**	**CoP Membership (N = 40)**
International organisation [INTORG] (n = 16)	Medical sciences (n = 21)	<10 years (n = 6)	Yes (n = 30)
National government (SSA countries) [NATGOV] (n = 5)	Economics (n = 11)	>10 years <20 years (n = 20)	No (n = 10)
Academic institution in SSA countries [ACADINST_AF] (n = 3)	Other social sciences (n = 4)	>20 years (n = 9)	
Academic institution in Europe [ACADINST_EU] (n = 2)	Other health sciences (n = 4)		
Independent consultant based in SSA [INDCONS_AF] (n = 5)			
Private for profit [PRIVFP] (n = 4)			
Private not-for-profit [PRIVNFP] (n = 4)			
Other [OTHER] (n = 1)			

Abbreviations: SSA, sub-Saharan Africa; CoP, community of practice.

**Figure 1:**
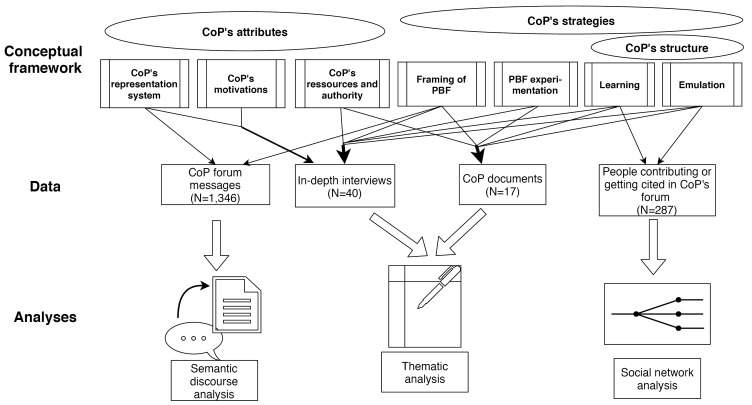
Correspondence Matrix Between DE Framework Dimensions, Quantitative and Qualitative Datasets, and Analyses. Abbreviations: DE, diffusion entrepreneur; CoP, community of practice; PBF, performance-based financin.

Interview transcripts and CoP-related documents were coded using QDA Miner©. We applied a prominently deductive approach based on the DE framework dimensions. All authors reviewed and approved a preliminary codebook prior to completing the coding. Thematic analysis was subsequently applied to the coded data. Figure 1 shows how each dimension of the DE framework connects to the different datasets and analyses.

Upon completion of analysis, using an interactive strategy of merging,^27^ we brought the sets of quantitative and qualitative findings together through a combined analysis. For each of the DE framework dimensions outlined in Figure 1, we compared and synthesised the related qualitative and/or quantitative findings on the CoP’s attributes (ie, representation systems, motivations, resources and authority), its network structure, and strategies (ie, for inducing PBF framing, policy experimentation, and emulation and learning). In the results section, we employ a narrative approach whereby findings are presented along each dimension of the framework.

## Results

### Emergence and Evolution of the Performance-Based Financing Community of Practice

**Figure 2:**
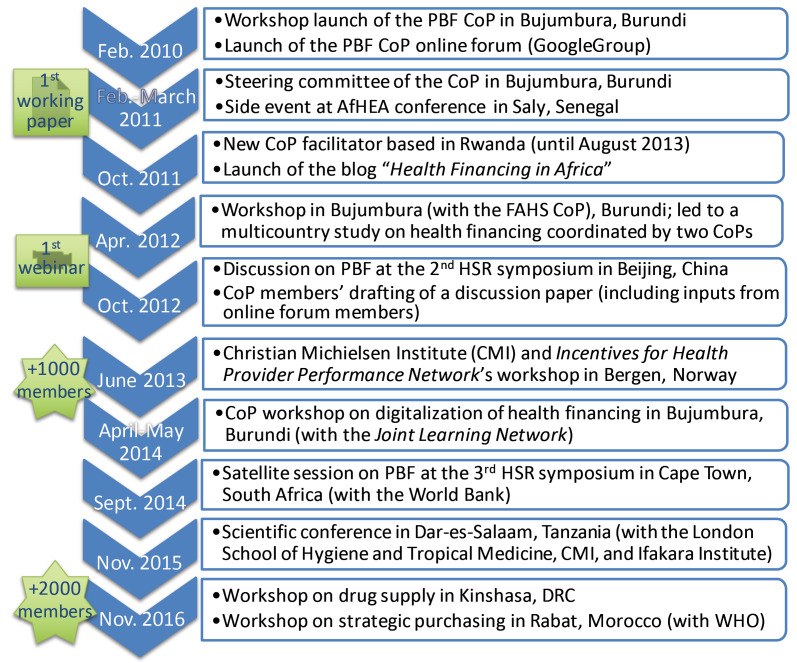
Overview of the PBF CoP’s Milestones and Activities, 2010-2016. Abbreviations: AfHEA, African Health Economics Association; CoP, community of Practice; FAHS, financial access to health services; HSR, health systems research; PBF, performance-based financing; DRC, Democratic Republic of the Congo.

Conceived as a transnational knowledge exchange network, the CoP brings together a community of practitioners who (*i*) share a common interest in developing and sharing PBF information, and (*ii*) promote a community of PBF experts engaged in fostering the diffusion of PBF in SSA.^28^ By the end of 2016, most of the PBF CoP’s 2000+ members were Africa-based consultants, health workers, and policy-makers.^29^ Figure 2 represents the evolution of the CoP, featuring major events and face-to face or online activities in which the CoP played a leading or influential role.

Among the CoP’s key events, the main facilitator asserted that the founding moment was the 2010 workshop in Bujumbura:

*“Everything’s there actually, in this workshop. The whole concept is there. […] How... we put African experts forward, […] the joint organisation with the Ministry of Health with a very open model, putting forward young researchers... So, actually... we’re breaking with conventions. […] This founding workshop is one of the best representations of what we wanted”*(I19a_ACADINST_EU).

The founding workshop notably gathered the CoP 6 core group members (ie, the CoP inner circle, of which 4 members came from high-income countries [HICs]). For the core group, such “breaking of conventions” involved diversifying knowledge exchange formats and convening hybrid types of workshops, gathering academics, development experts, and practitioners. The Bujumbura and Dar-Es-Salaam events in 2014-2015 were typical examples of such workshops, jointly organised with other institutions and featuring strong CoP participation.

The CoP online forum was created shortly after the founding workshop. The number of members soon exploded (see Figure 2), thereby contributing to the expansion of its influence. Many informants from major global health organisations recognised this influence. Table 4 includes information about the PBF topical discussions on the forum, including on participation, number and nature of citations, and members’ influence.

**Table 4:** Information About the PBF CoP Online Forum Participation, 2010-2016

**Participation**	287 CoP members posting in topical discussions	68.1% (LMICs), 66.2% (SSA)
**Citations in 1346 topical posts**	Among those citing (N = 186): 63.9% cite >1 person 49.5% get cited	Among those cited (N = 215): 81.9% (CoP members) vs. 18.1% (non-members) 52.6% (LMICs), 51.2% (SSA)

Abbreviations: CoP, Community of Practice; LMICs, low- and middle-income countries; SSA, sub-Saharan Africa; PBF, performance-based financing. *Note*. Non-member: someone outside of the CoP network.

### The Community of Practice’s Attributes, Structure, and Strategies to Induce Policy Diffusion

#### The Community of Practice’s Attributes: Representation Systems

**Figure 3:**
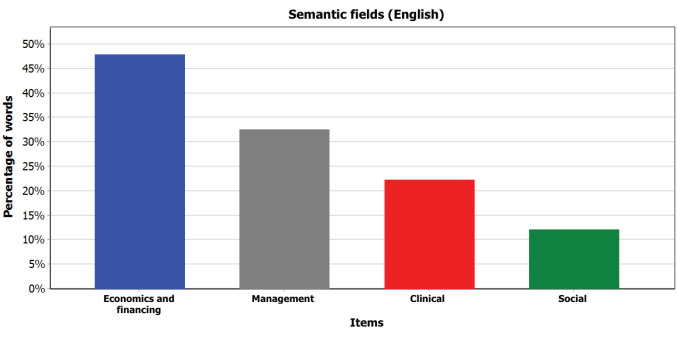
Major Semantic Fields in English Discussion Threads.

**Figure 4:**
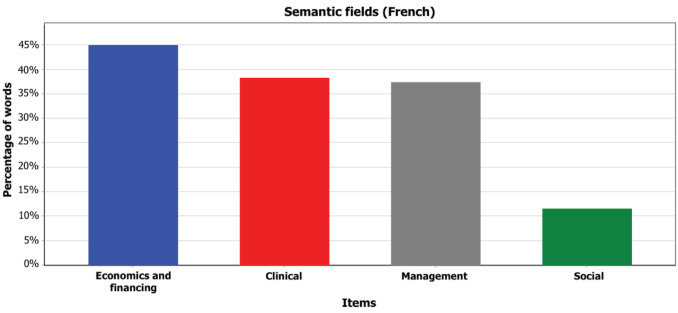
Major Semantic Fields in French Discussion Threads.

The CoP members’ representation systems transpired through the discourse (ie, choice of words, expressions, and metaphors to describe and/or comment on PBF discussion topics) used in online discussions. The prominence of economics and health/clinical semantic fields (Figures 3 and 4) reflected, ie, in economics and later specialising in health (especially for English-speaking members), or in health/clinical sciences complemented with a degree in economics (especially for French-speaking members). The “economics/financing” semantic field dominated both English and French discussions. Social sciences and social themes were less prominent. Influential members were keen on developing a PBF language that spoke to the CoP members’ training background.

Based on interview data, we found that core group members shared a common history (eg, participating in initial PBF pilot scheme implementation), and additional members had been exposed to core members during training (in Europe) or pilot scheme experimentation. This shaped a common language. Informants mentioned that from the beginning of the CoP’s activities, people’s background was indeed fundamental to developing the “idea of a club.” This feature might explain the discussions of problem representations using a common professional jargon on the online forum.

This analysis thus points to a rather cohesive community of economists, health workers and managers.

#### The Community of Practice’s Attributes: Motivations for Mobilising the Community…and Promoting Performance-Based Financing

Analyses of interviews with core members and documents enabled us to identify 2 key interests in mobilising this community, which are intertwined with interests for promoting PBF: (1) gaining political influence and market space on the global health policy-making arena by fostering a legitimate discourse shaped by hundreds of LMIC/African practitioners; (2) consolidating the body of lay knowledge on first-hand PBF experiences to build a shared repertoire of resources that would inform the development of PBF definitions and standards (eg, the PBF Toolkit^23^) highlighting that PBF is indeed a key policy solution. All CoP members pursued both ‘genuine’ interests in developing knowledge on how to improve health systems reform design and implementation (through experimenting and learning from PBF), and self-regarding motives.

For HIC members, the first motivation translated into the CoP’s positioning, ie, putting SSA practitioners and their discourse forward. Informants belonging to international organisations perceived this endeavour as laudable and successful:

*“For me, well, [the CoP] was effective in the sense that today, finally, what I see is that it’s often much more... African practitioners who work on PBF than... those from the World Bank...”*(I20a_INTORG).

According to several informants, some of CoP core members used the CoP’s image – promoting SSA practitioners and their framing of PBF – to disrupt hierarchies in the political economy of global health policy-making, and to increase their visibility. SSA members’ gains could be reputational/political (expanding influence in home country), financial (higher salaries), and professional (career advancement and knowledge/skills expansion). Indeed, the PBF CoP enabled access to diverse job and training opportunities. But SSA practitioners also had a lot to gain from promoting PBF itself (eg, gaining wider visibility in the global health arena), by openly sharing their lay experience of the policy using the CoP forum and blog.

The second motivation, which featured the core group in particular, implied promoting the fundamentals of PBF. One CoP member considered that the priority agenda remained that of promoting a PBF standard definition:

*“In the definition of a CoP […] there’s an important component, which is the strive*(pause)* to find local definitions, local solutions. Yet, in*(name removed)* his slides, you won’t see this part of the definition. […] I don’t know [why]. But when you look at how he understands PBF, for him it was never intended as a tool to invent local solutions”*(I34_ACADINST_AF).

The idea of upholding and advertising a community of local practitioners did not involve going as far as to promote local solutions for health systems originated by the latter.

#### The Community of Practice’s Attributes: Resources and Authority

One of the major strengths of the CoP network was that the core group members had very strong social resources, which enabled them to build personal relationships with health financing experts from LMICs. Even prior to launching the CoP, Europe.

Besides social resources, core group members also enjoyed crucial political resources: they had often previously interacted with influential politicians. This facilitated access to them. Core group members also had extensive knowledge resources, which they readily transmitted to CoP members. Informants notably asserted the CoP’s effective contribution to the emergence of “*African champions […] who have become leaders in PBF, […] providing the technical support and knowledge around PBF from within their countries*” (I53_INTORG). Many member and non-member respondents highlighted the capacity of the CoP’s most influential members (mostly coming from HICs) to inspire and “mould” SSA PBF experts. Using the CoP, the core group coached SSA experts, to the point that the latter have reportedly “*overcome the masters*”(I53_INTORG).

These resources and prior work experience prompted core members to gain prestige, which helped them exert expert and scientific authority on the global arena. To realise its ambitious agenda, the CoP also needed some material resources. Across the years, the facilitation team secured occasional funding from The World Bank, the African Development Bank, and the Rockefeller Foundation. A Norwegian Institute, Cordaid, and a consulting company also financially contributed to specific CoP activities.

#### The Community of Practice’s Strategic Framing of Promoting Performance-Based Financing

One of the explicit goals of the CoP was to formulate a clear vision and definition of PBF:

*“We need to define better what we mean by this ‘PBF’ approach. The experience that we developed in Rwanda, whose lessons are being applied to Burundi, and to Zambia […]. This is the story. It needs to be written up”*(I16, forum post).

This was made possible through the sharing of common problem representations structured by initial training (see above) and, according to most informants, SinaHealth courses which almost all SSA members completed prior to joining the CoP. Concurrently, in members’ posts, PBF was mentioned in relation to health planning and financing, increasing efficiency of public spending, and autonomy of health providers. Influential CoP members developed a specific PBF jargon shaped by these semantic fields (eg, “purchasing agency”), and used words pertaining to the private sector (eg, “business plan”). Such shared repertoire (a key ingredient in the CoP’s success) using a very specific technical language shaped a strong collective identity. Some informants portrayed this repertoire as having normative overtones, while others, more critically, depicted it as a “doctrine.”

PBF made sense for SSA practitioners because it matched their representation systems: a lot of them considered that in the *status quo* (ie, input-based financing) “*money was being wasted*” (I54_INDCONS_AF). The situation called for alternative ways of acting:

*“[PBF] creates a spirit of entrepreneurship... People start... thinking out of the box, people stop thinking under the usual constraints […] So it’s a real paradigm shift”*(I26_INDCONS_AF).

CoP members thus portrayed PBF as both a revolutionary and pragmatic solution. Such depiction may also explain the wide consensus in online discussions. Dissonance across members emerged only in 3 discussions: once about user-fee exemptions, once about privatisation of drug provision, and once about health managers’ spirit of entrepreneurship. This framing also contributed to the portrayal of PBF as a legitimate solution because it had been co-produced and propelled by SSA practitioners, who represented, as shown in Table 3, two-thirds of CoP participants. In coherence with the core group’s first motivation outlined above, the co-production of PBF framing thus appeared to have a legitimising effect – for the CoP and for PBF itself. However, some interviewees voiced concerns as to whether co-production actually took place. The PBF definitions referred to by SSA members typically were those developed by non-SSA members.

#### The Community of Practice’s Strategic Contribution to Policy Experimentation

The first challenge in PBF diffusion was “*to provide governments and their partners with the appropriate technical assistance.*”^28^ While agreeing that PBF “*was going to be something very big*” that would “*conquer the continent*” (I19a_ACADINST_EU) global experts in the late 2000s expressed concern that capacities were lacking in SSA. The CoP emerged as a tool to create an enabling environment for smooth policy experimentation:

*“The CoP was a strategy for me to... reinforce technical capabilities. […] Strengthen the quantity [of African experts]... and make sure these experts are real experts”*(I19a_ACADINST_EU).

First, the CoP’s technical and “Africa-led” framing of PBF nicely matched this endeavour: by bringing together a critical mass of well-trained SSA practitioners, the CoP offered international organisations and companies (funding and/or implementing PBF pilot programmes) a pool of SSA experts to tap into:

*“[The CoP] was a big vehicle to make sure […] that … you had a roster of people who were involved in PBF projects or interventions who could potentially be… further … trained and coached… to become the experts they have become!”* (I53_INTORG).

The CoP’s facilitation team connected job announcers to SSA PBF experts. Soon, the CoP became the indispensable “*market place*” (I19a_ACADINST_EU) for PBF experimentation, since it enabled consulting companies to post their call for tenders and job offers, while encouraging SSA members to apply. These jobs were typically located in PBF experimentation settings. The CoP therefore directly contributed to exporting practitioners to other SSA countries. This pattern further expanded through the action of a separate, yet connected entity: SinaHealth’s training company and its alumni network (reportedly also influencing continental diffusion processes). The latter trained multiple country teams starting to implement PBF. At the end of each two-week course, alumni would systematically be invited to join the CoP. The training score was used to “vouch” for CoP members’ expertise in their job applications. Informants described the following sequence: people were trained by SinaHealth, joined the CoP, engaged in knowledge exchanges and study tours, gained visibility, and started diffusing the above-mentioned repertory of practices. Such repertory was primarily shaped by first generation DEs, ie, CoP members from HICs (including SinaHealth’s head) on PBF in other experimentation settings. This sequence is referred to as the “second-wave DE” phenomenon.

Second, through the online forum and other CoP activities, CoP members facing challenges in introducing and/or implementing PBF were provided technical advice and guidance. Informants indeed perceived the CoP as a highly relevant tool for fostering policy experimentation, by supporting PBF practitioners’ work and career advancement across the continent. This network thus represented a strong catalyst for diffusion processes through its multifaceted support of policy experimentation.

#### Community of Practice’s Structure and Strategic Inducing of Policy Emulation

Bringing pilot experimenters together naturally attached social value to the tested policy, thus increasing PBF legitimacy and inducing policy emulation. Yet, endogenous processes occurring within the CoP reinforced policy emulation as well. Specifically, the cohesive structure of the CoP network and the nature of social interactions among members featured a strong sense of belonging to the CoP.

**Figure 5:**
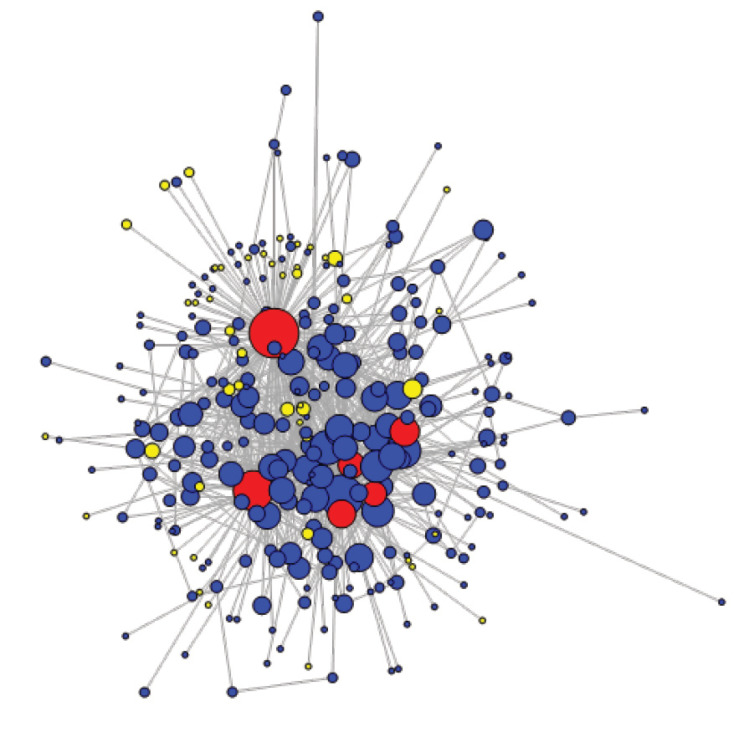
Weighted Representation of the CoP Thematic Discussions’ SNA, 2010-2016. Abbreviations: CoP, Community of Practice; SSA, sub-Saharan Africa; SNA, social network analysis.

CoP members’ cohesion primarily built on a shared appreciation that PBF was to be promoted. This was made possible by implementing emulation strategies. The CoP endeavoured to spark “*collective enthusiasm*” (I19a_ACADINST_EU) by creating multiple avenues for practitioners to socialise with one another. The CoP notably supported study tours for members to engage in inter-country exchanges, thereby fostering network cohesion. The SNA provides a rich visualisation of the network’s cohesive structure. The giant size of the strong component (Figure 5) shows that the network of CoP members’ citations in the forum is indeed highly connected. Although citing fellow CoP members does not necessarily mean being actually connected to them, at least it features a sense of belonging to the same online community. Core group members are featured in red; non-members in yellow.

The network’s global clustering coefficient is 0.098. This coefficient indicates that, on average, there is a nearly 10% chance that 2 individuals, who are citing or cited by a common individual, are also connected to each other. This coefficient can be considered average.^31^ More salient is the average path length of the network (ie, the mean shortest paths between all pairs of nodes) of 2.82, which suggests that the CoP is a “small world,” ie, with fewer than 3 people separating each node. We tested the clustering coefficient and average path length of the CoP network against those of 1000 random networks (using Erdös-Rényi’s model), and found that the CoP network indeed qualifies as a “small world.”^31^ This analysis of participation in the CoP topical forum discussions appears to confirm the community’s strong cohesion.

**Figure 6:**
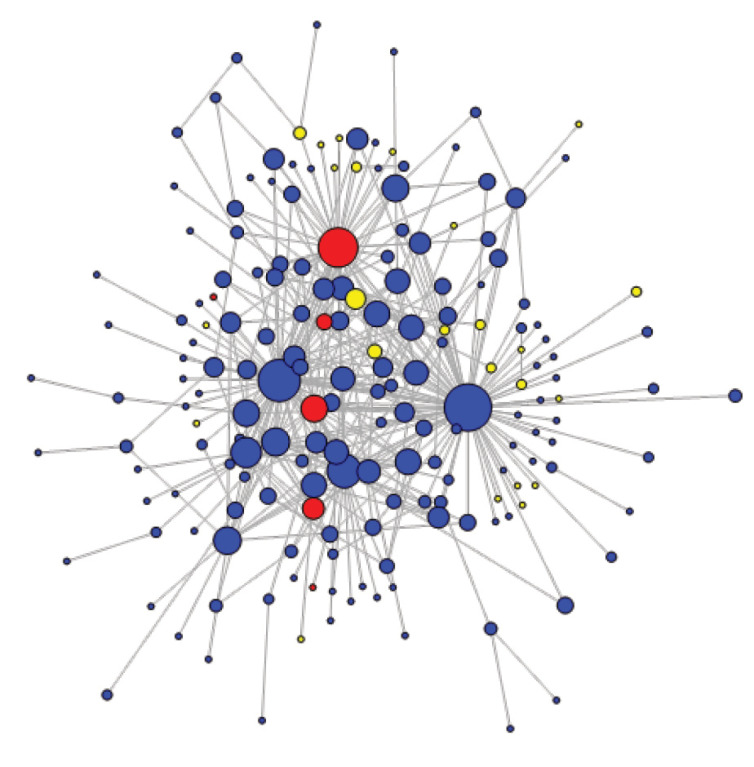
Weighted Representation of the CoP Online Thematic Discussions’ SNA, 2010-2012. Abbreviations: CoP, Community of Practice; SSA, sub-Saharan Africa; SNA, social network analysis.

**Figure 7:**
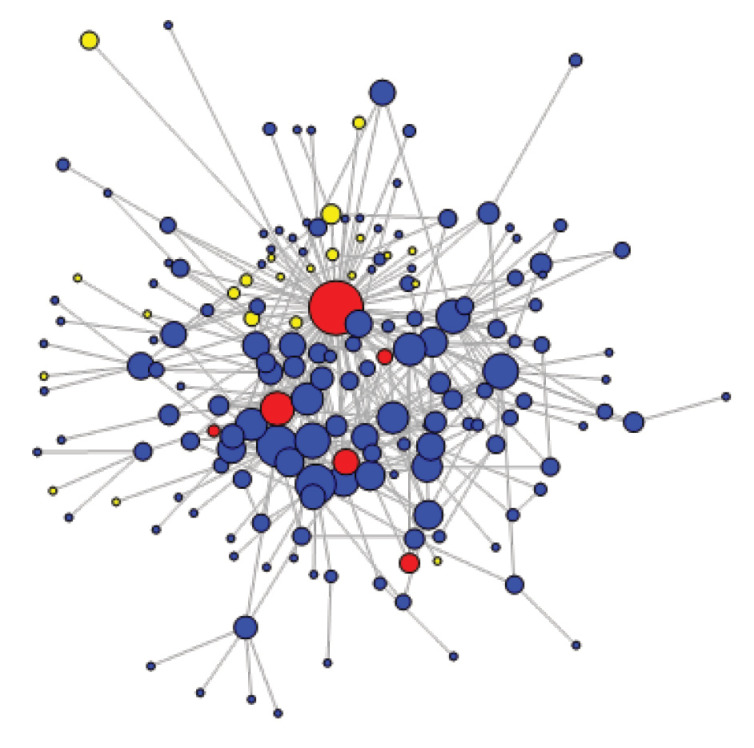
Weighted Representation of the CoP Online Thematic Discussions’ SNA, 2013-2016. Abbreviations: CoP, Community of Practice; SSA, sub-Saharan Africa; SNA, social network analysis.

Figures 6 and 7 illustrate the evolution of the network structure over time. Average path length was shorter in earlier years (2.78) compared to more recent ones (3.06). The global clustering coefficients are constant through time (10.8%). This temporal analysis shows that the network was more active and more cohesive in the early years than it was in its last 3 years, as confirmed by the informants’ general perception. For example, members sharing their emotions on the forum (eg, following the death of a fellow member) occurred more often in 2010-2012.

The SNA also enabled us to visualise members’ influence (Figure 5), measured by weighted degree centralisation (NB: for figures 5-7, size of the nodes is proportional to weighted degree centralisation values). The member with the highest weighted degree (also the one that is represented as the largest node) was the CoP main facilitator (352). Although he was not a core group member, the individual with the second highest weighted degree was SinaHealth’s head (160). These 2 highest degree nodes remained constant over time. This is coherent with qualitative findings, which highlight a consistent cross-fertilisation between the 2 major PBF networks in SSA. In fact, several informants suggested that the influence on diffusion of SinaHealth’s network and the CoP would be very difficult to disentangle, given their very close interaction.

Still, when looking at influence in terms of generating inspiration and career aspirations for SSA individuals, the CoP more often than the training company. Policy emulation inferred from the CoP’s activities built on a strong community feeling emerging from the solidary nature of members’ interactions. Informants reiterated the CoP’s instrumental role in harnessing a critical mass of practitioners sharing and supporting the same policy idea:

*“One of the major forces [of networks] is that very quickly you have a large number of people who seem to support the same concept.”*(I34_ACADINST_AF).

Core group informants portrayed the CoP as a community that “*enabled [isolated members] to gather together and identify with it*” (I17a_PRIVFP). For a CoP member in a given SSA country, the fact that “*dozens of other*” CoP members were available at country level to support him indeed had a de-isolating effect. This would in turn foster policy diffusion: local policy-makers – whom CoP members were reportedly socialising with – would be more keen on listening to a policy idea when it was supported by numerous trusted people.

Other informants, however, voiced concerns that policy emulation across experts was not exactly Africa-owned, but rather relying on and still building on HIC members’ expertise. The branded “horizontal” nature of the CoP was questioned.

#### The Community of Practice’s Structure and Strategic Shaping of Policy Learning

Lay knowledge was generated and shared through multiple interactive activities (including an online forum on best practices, an online readers’ club, face-to-face workshops) and using various formats (blogging, working papers, webinars, scientific papers). Members shared success stories.

Among the various learning activities, informants tended to agree that face-to-face activities were the most powerful learning tool. According to one of the facilitators, the CoP did not manage the entire learning process of its members aspiring to become international experts.

By valuing lay knowledge, the core group shaped an action-oriented type of learning agenda, so as to support each phase of PBF policy experimentation, from small-scale pilot to national rollout. This form of agenda was preferred to an academic learning agenda so as to “*leave space for enthusiasm*” (I19a_ACADINST_EU). Coincidentally, informants feared that there was not enough “quality control” of the validity of knowledge that was shared. The main facilitator reckoned he lacked time to perform quality control. In fact, we noted that in the forum, CoP members rarely pointed to academic papers to contradict or nuance a practitioner’s argument that might be considered scientifically invalid by researchers. The fact that many SSA practitioners were expecting to gain professional recognition from participating in CoP activities is likely to have influenced the nature of their contribution. However, the funders’ participation in the online forum was considered fairly low – aside from posting job announcements.

**Figure 8:**
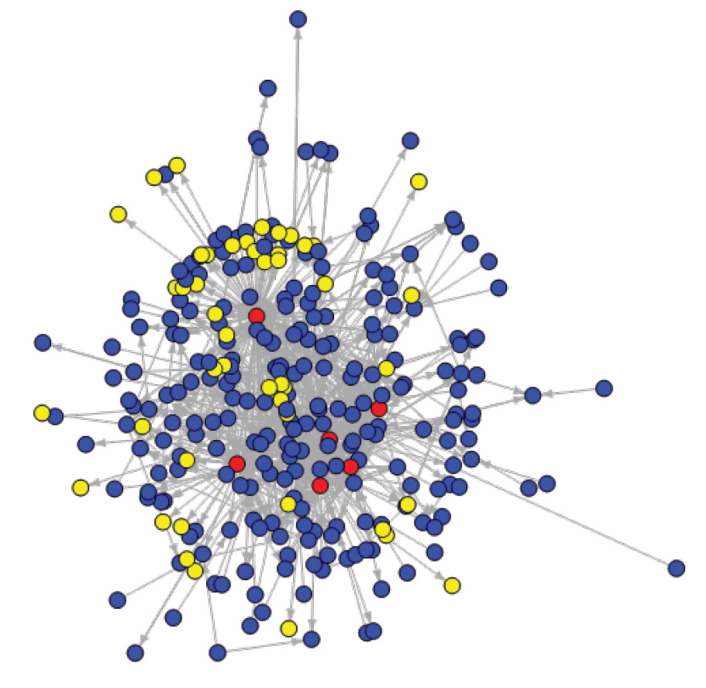
Oriented Unweighted Representation of the CoP Online Thematic Discussions’ SNA, 2010-2016. Abbreviations: CoP, Community of Practice; SSA, sub-Saharan Africa; SNA, social network analysis.

Core group informants also emphasised the idea that technology advancements and ability to travel more easily across countries were enabling factors for implementing the learning agenda. Putting forward local practitioners translated into CoP processes through citing members during face-to-face workshops and in online activities. These activities were indeed made possible by technology and globalisation. Contributions to the CoP online forum may have served as a proxy for personal influence, because it would give visibility to individuals. In this context, citing fellow CoP members represented an implicit recognition of that person’s contribution to policy learning on PBF. The oriented modality was used in Figure 8 arrows, to feature the direction of the links (citing or getting cited). Cited non-members care featured in yellow.

Of those cited (N = 215), 18.1% were non-members (and 43.5% of those are only cited by the facilitators). As explained above, the main facilitator strategically conceived a hybrid model of face-to-face workshops, gathering both academics and SSA practitioners (see examples in Figure 2) and engaged himself and fellow CoP members in regular blogging, to “*build bridges with the outside*” and “*impede some sort of complacency*” (I19c_ACADINST_EU). Despite these efforts, SNA results indicated that the CoP remained a mostly inward-looking learning community. Besides, most commonly cited non-members are authors of academic papers highlighting mixed evidence on PBF (eg, ^32^). Non-members’ authoring papers featuring positive evidence about PBF were also cited in forum discussions, but their mention induced shorter debates (hence, a lesser number of citations). This result might reflect limited openness to PBF criticism and a tendency towards confirmation bias. Concurrently, several interviewees mentioned that a common defensive tone was often used in online posts in reaction to externally-voiced criticism of PBF (including in scientific publications) and the CoP process itself.

Looking at the number of citations from 2010 to 2016, we found that SSA members represented only 41.5% of the total number of citations, while they posted 66.2% of the total number of online messages. This would mean that SSA members do not get cited as much as they participate. If we consider that someone being cited means that he/she has knowledge influence, then HIC individuals are the ones driving the learning agenda. None of the authors of a scientific discussion paper^33^ which was – amongst others – fed by comments from CoP members and discussions during Bergen’s workshop (Figure 2), was based in SSA. Still, analyses of cited people over time enabled us to nuance influence patterns between HICs and LMICs (mostly SSA). The number of citations of LMIC/SSA members (of the total number of citations) increased from 34.6% to 52.1%, indicating a growing African ownership of policy learning.

Lastly, CoP’s empowering SSA expertise sparked similar autonomous processes in other major PBF actors such as the World Bank. Several informants from that organisation indeed acknowledged being inspired by the CoP’s model for promoting practitioners and having them travel throughout the continent. This soft influence also contributed to the portrayal of the CoP as the catalyst DE in SSA, and enhanced policy learning. The most salient illustration was the development of a culture of “importing-exporting” SSA experts for the purpose of helping a country team learn from the expertise of a foreign consultant. The World Bank’s participation in the making of second-wave DEs served to increase the chances of success of a PBF pilot programme.

## Discussion

This paper offers in-depth and nuanced accounts of how the CoP became the catalyst for PBF diffusion processes in SSA. Drawing on our analysis of its attributes, structure and strategies, 4 key findings illustrate how the CoP reached this status: first, through implementing its agenda to define and disseminate a common repertory of PBF practices framed as country-driven and appropriated by SSA practitioners; second through its multifaceted support for policy experimentation and learning; third through its perceived capacity to spark policy emulation and empower career advancement for SSA experts; and fourth through its model of promoting practitioners and having them travel throughout the continent. This soft influence fostered major actors’ recognition of the CoP as one of the main catalysts for DEs in SSA in the mid-2010s. These key findings can be summed up in the unique phenomenon of “making second-wave DEs.” In closing the Discussion section, we critically reflect on the empirical and theoretical implications of this phenomenon.

### A Hierarchical or Horizontal Network?

The CoP operationalised its vision of a horizontal network through the development of multiple participatory and interactive learning activities. Yet this was not enough to change the governance structure of the network. Characterising a policy network involves looking at the “distribution of capabilities over the set of actors” within the network.^10^ Our analysis of the CoP’s resources and authority suggested that the most influential CoP members (mostly from HICs) were the leading actors fostering diffusion processes. Building on their initial PBF experience, they designed a learning agenda that strategically co-developed lay knowledge featuring and acknowledging the (African) context within which policy-making occurs, as in.^34^

In general, CoP members framed their activity as horizontal, apolitical and based on peer knowledge exchange (ie, as knowledge networks). However, as our analysis of the CoP proceeded, it became clear that the community in fact conveyed a major political project, ie, diffusing a policy (in this case, PBF). We can speculate that, for the sake of effectiveness, this project entailed ‘re-introducing’ a hierarchical structure of governance. Indeed, following Adam and Kriesi’s typology,^10^ the CoP fell under the category of a “hierarchical cooperation” type of network, ie, cooperation “conducted under conditions significantly different from those obtaining among actors all of whom are more or less equally powerful.”^10^ This unequal distribution of power was also featured in the SNA results. The promoted SSA-driven community did not match the network’s structure, which was dominated by HIC individuals.

Still, temporal analyses highlighted a growing SSA influence in knowledge exchanges. This indicates that the CoP’s governance allowed for a much better representation of LMICs than global policy-making arenas.^35^ Thus, in terms of inclusiveness,^36^ the intense participation of SSA practitioners in the CoP was one of the strongest legitimating factors contributing to PBF diffusion, because it proved that voices from LMICs were actively supportive of PBF. Yet, as identified in another study,^8^ it is unlikely that CoP members, being mostly health practitioners, international consultants, and national policy-makers, could be deemed legitimate representatives of SSA populations.

### Disrupting the Political Economy of Global Health Policy-Making?

Our study found that the CoP was perceived as offering an opportunity for SSA practitioners to gain political influence within the global policy-making arena, largely dominated by HIC actors (international organisations and the scientific community). Yet, according to most informants, the net beneficiaries of this particular endeavour were the most influential members, mostly coming from HICs. Ironically, the CoP contributed to legitimising PBF at the global level and with external audiences, precisely because it represented the voice of SSA practitioners.^8,16^

The assumption that major “stakeholders are more likely to interact with actors who are perceived as influential—independent of their beliefs—than with actors who are perceived as not influential”^37^ might be a barrier to the impact of policy networks with horizontal modes of operation. In other words, dominant organisations and individuals would be keener on listening and negotiating with peer-dominant HIC individuals.

Moreover, the ways the CoP effectively participated in PBF African experts’ empowerment were unclear. In fact, most SSA practitioners remained dependent on individual experts from HICs for professional and reputational recognition, and on funders (eg, PBF donors) for job opportunities and financial sustainability. This feature reflects the persistence of socio-historical structures and representation systems that continue to value the “white expert” above the local expert. The recent migration of the CoP to Collectivity’s web platform may offer more diverse opportunities for experts to engage in a more decentralised governance of the community.

In addition, some individual DEs coming from HICs were genuinely keen on promoting African experts’ creative ideas for adapting or enhancing PBF design characteristics (eg, the SinaHealth coursebook^38^ features some of the home-grown ideas to implement PBF). Yet, this study’s findings highlighted that, in their view, the core PBF fundamentals deserved precedence over local adaptations. In other words, promoting a repertoire of PBF practices that featured their own representation systems was perceived as more important than effectively empowering SSA experts. This perspective is coherent with findings from a large study on knowledge transfer, which highlights differentiated valuing of expert knowledge and ideas relative to policy actors’ positioning in global governance.^39^

### An Epistemic Community With Strongly Intertwined Characteristics

Similar training backgrounds and a common history (eg, attending SinaHealth courses) consolidated SSA CoP members’ sense of belonging to a community, thereby reinforcing policy emulation and spurring a shared identity: being PBF practitioners. The CoP was indeed portrayed as a transnational community of “professionals with recognized expertise and competence in a particular domain.”^40^ Hence, it would qualify as an “epistemic community” despite its hierarchical configuration. The sense of fraternity also built on citations in forum messages: members citing fellow CoP members created a sense of being part of the same policy community.

Moving past a mere analysis of the CoP’s structure, interests, ideas, power (resources/ authority), and strategies, we tried to unravel the ways these features are intertwined to foster diffusion. Shiffman suggested looking at how “historical precedent and structural forces interact with individual and organisational agency”^2^ to advance policy diffusion. The CoP’s interaction with other PBF DEs (eg, SinaHealth, The World Bank), and the fact that its most influential members were individual DEs who had worked together in the past, significantly contributed towards fostering the impact and perceived legitimacy of the CoP.

### A Normative Inward-Looking Community?

The norms promoted through a shared language and positioning towards PBF had both positive and negative implications for the policy network.

First, although the CoP had a great diversity of participants in terms of position/institutional affiliation, the fact that they shared a similar language and PBF experience made them more likely to welcome and participate in internally generated knowledge (ie, lay knowledge shared by fellow CoP members) than externally generated knowledge. Our findings were thus consistent with the hypothesis that nodes in a community were more likely to connect to other members of the same community than to nodes in other communities. This is coherent with findings about the structure of health policy networks in Burkina Faso,^41^ which showed that strong cohesion and shared norms are often considered barriers to innovation.

Second, such homogeneity in turn implied that internal knowledge would be less likely to be contested than evidence produced by non-members. Bertone et al^7^ pointed to the risk that CoP members “overestimate” the external validity of their lay knowledge. Despite the facilitating team’s efforts to open up debates (eg, by inviting academics to CoP workshops), we found evidence that CoP members sometimes used a prescriptive tone in knowledge exchanges. This tone may also have to do with members being cognisant that their contribution to CoP activities might generate career opportunities offered by CoP’s partners.

Third, in the early years of the CoP, promoting practitioners’ lay knowledge on PBF involved opposing it to academic evidence. Interestingly, members’ cohesion built on this defensive language; and this in turn consolidated a sense of community. Members’ efforts to legitimise lay knowledge also involved opportunistic citing of academic evidence on PBF produced by fellow members. Thus, policy learning was effectively featured through members’ citations: citing fellow members involved recognising that this person’s contribution expanded PBF knowledge.

### Empirical Implications to the Identification of Second-Wave Diffusion Entrepreneurs

Another key contribution of this paper is the identification of second-wave DEs. This category is distinct from existing terminologies to represent local actors involved in policy diffusion, such as “national champions.”^42,43^ Indeed, unlike in these studies, second-wave DEs were initially coached by HIC individuals with whom they had worked or interacted in the past (eg, in training/academic settings). First-generation DEs carefully “chose” these SSA practitioners and endeavoured to propel their career to the next level, provided that the latter have proven expertise in the promoted policy and willingness to support it at home.^18^ This phenomenon is also reflected in SNA results, which highlight a how the number of cited SSA CoP members grew over time, suggesting increased recognition. This process therefore reflected and reinforced policy emulation and learning, with first-wave DEs (ie, those from HICs) inspiring and grooming SSA DEs. Some of them reportedly turned into leaders “*overcoming their masters*,” engaged to diffuse PBF in their home country and beyond. As an illustration, one very active CoP member (from Rwanda) founded their own consulting company to provide advice to pilot PBF project implementation teams across the continent. The making of these experts involved all levels of classic political hierarchy (from the decentralised to the global level) and transcended borders between public and private actors on the one hand, and decision-makers and experts on the other hand.^44^ As such, it proved a vivid illustration of polycentrism^14^: our findings invite further analysis of transnational diffusion processes involving a configuration of multiple autonomous units of political influence.

The making of second-wave DEs must also be considered in all its complexity and nuances. On the one side, the process served global-level DEs’ interests (ie, their framing of a PBF policy led by African experts). On the other side, these global-level DEs are those who, in most cases, validate the legitimacy of SSA experts at global level (through training and multiple forms of expertise promotion), and who finance their career advancement. In this context, the actual redistribution of power between HICs DEs and DEs from LMICs appears to be quite limited. In fact, at country level wave DEs’ actions were at times limited, notably in terms of influencing national policy-makers. Two reasons may explain this outcome. First, they might have been perceived as less credible or renowned than their Western peers. Second, they remained vulnerable to political turnover.^18^ Therefore, despite the successful example outlined above, the idea, readily promoted by many global-level DEs, that PBF diffusion empowers second-wave DEs, can be challenged. We would be very interested in seeing future research investigating both the second-wave DE making processes and the forms of power emerging from the interaction between global- and national-level DEs in this making.

### Fostering Policy Emulation Rather Than Actual Policy Diffusion?

Findings from this paper provide food for thought to scholars reflecting on North-South policy diffusion. Global-level DEs’ strategy of valuing and going through local intermediaries (ie, individual experts and Africa-based companies) to secure key high-level actors’ buy-in seemed to yield positive effects in terms of policy diffusion (at least, in appearance – when looking at the PBF diffusion rate in SSA^16^). This finding suggests that, from the perspective of those actively engaged in policy diffusion, the promotion of SSA practitioners grant more legitimacy and ensure greater efficiency in policy diffusion, than the simple North-South policy transfer. Discursively, this legitimation process strategically matches the popular language of Africa’s participation in global health policy-making.^45^ Because such “local-making” is funded by influential global health governance actors, this suggestion may illustrate the “moral resurrection of aid,” which emphasises locally-owned, participatory South-driven processes.^46^ Future quantitative and qualitative research — including outside the field of global health — should further examine the successful making of DEs acting at the continental and (sub)national levels, and their impact on policy diffusion outcome(s).

However, one may ask whether such outcomes actually relate to actual policy diffusion. Among all policy diffusion mechanisms,^12^ policy emulation was featured most prominently in our study. Results shed light on both the strong community feeling emerging from participation in the PBF CoP online forum, and the “mass effect” that led to de-isolating its members. Indeed, the CoP model primarily relied on the transfer of technical expertise from North to South and replicated by SSA practitioners. This included agreeing on a common framing of PBF (ie, an Africa-driven policy solution), facilitating policy experimentation (eg, developing PBF best practices guides), and fostering policy learning (by promoting inter-country and peer-to-peer lay knowledge exchanges). Global-level DEs conceived all these activities in the hope that they would facilitate PBF diffusion in SSA countries, while promoting, coaching, and creating African experts. Because strategies of policy framing, experimentation, and learning were largely controlled by HIC DEs (as our mixed method study findings highlight), all of these policy diffusion mechanisms essentially and ultimately served to foster policy emulation, as suggested in.^16^ Given the central role of policy emulation (due to the prominence of global-level DEs) in the present study of PBF, we encourage policy diffusion scholars to further look into the sustainability of (apparent) diffusion outcomes, asking the question: does the number of governments who have engaged in implementing a given policy remain stable over time?

### Study Strengths and Limitations

The context of increasing polycentrism in global governance makes it critical to shed light on the role(s) played by new transnational actors, such as policy networks including CoPs. This empirical research enabled us to identify the critical dimensions of CoPs facilitating the diffusion of health system reforms in polycentric governance. Semantic discourse and SNAs helped to unfold networks’ characteristics, ie, their constituting features (nature), structure, and strategic actions (agency). To explore these intertwined characteristics of transnational networks in global health policy-making, further mixed method research combining such innovative tools data is needed.

Our research also had several limitations. Our (retrospective) approach also included little observation of the CoP’s activities. In addition, the categories we used for the semantic analysis coding was derived from a previous publication: this may have introduced a confirmation bias. In addition, although we managed to yield a representative sample of CoP members, there may have been additional motivations for practitioners to participate in the CoP that do not emerge from this study.

## Conclusion

Our research brought to light the main attributes, structure, and strategies of a policy network – the PBF CoP– that appeared to catalyse continental efforts towards PBF diffusion in SSA. This study also showed that, despite good intentions to disrupt the established policy-making landscape, influential individuals and organisations from HICs continue to drive the framing and shaping of health systems policy experimentation, emulation and learning agenda, even if these are implemented in sub-Saharan African countries.

Our results also shed light on the complex social phenomenon of influential DEs’ making of “second-wave DEs” based in SSA. This thought-provoking phenomenon, which goes well-beyond the mere identification of national “policy champions” in LMICs, calls for additional theoretical developments and future empirical research related to other global health policies.

## Acknowledgements

This research would not have been possible without the significant amount of time that informants generously dedicated to answering interviews and emails initiated by the first author. We thank all the CoP members who voluntarily contributed to the research. Authors are particularly grateful to the lead facilitator of the PBF CoP for encouraging the research and commenting on an earlier version of the paper. This does not imply endorsement of findings or of their interpretations.

We also acknowledge the considerable support of Thérèse Gautier-Garancher, Raoul Funtchue Fongue, and Konan N’Guessan in interview transcriptions. Our thanks also go to the employees of the research NGO Miseli and in particular Tony Zitti, who helped with quantitative data compilation. We would also like to thank Guillaume Fournié for providing assistance in conducting our SNA, and Isabelle Guérin for her continuous support of this research. Lastly, we would like to thank Heather Hickey for proofreading the manuscript.

## Ethical issues

Prior to responding to the interviewer’s questions, all participants read a detailed information sheet and provided their written consent. Ethical approval was obtained from the University of Montreal’s *Comitéd’éthique de la recherche ensanté* (Certificate 16-153-CERES-D).

## Competing interests

VR was a co-researcher on the baseline study of the impact evaluation of PBF in Burkina Faso, but has received no salary from the funding body (the World Bank) for this activity. MDA is lead researcher on several process and impact evaluations of PBF in SSA countries, but she did not receive direct payments for any of those. The funders did not take part in the preparation or publication of this manuscript. The authors have no conflicts of interest regarding the publication of this paper.

## Authors’ contributions

All authors contributed to the design of the research. LG collected the qualitative data, transcribed verbatim 27 of the 40 interviews. With the help of a research assistant, LG extracted and compiled the data for social network and semantic analyses. LG performed the social network and semantic analyses. LG also coded and analysed the qualitative data, and drafted the first version of this paper. MDA and VR participated in several CoP events and aided in the data analysis. All authors contributed to the writing and reviewing of the manuscript and have read and approved the final manuscript.

## Funding

LG received a PhD scholarship from *Fonds de Recherche du Québec – Société et Culture* (FRQSC). LG was able to collect data in various foreign settings thanks to the generous support of Canada’s International Development Research Center (project ID#108038), the French Research Development Institute (IRD), and the Canadian Institutes of Health Research (Applicant #372369).

## Authors’ affiliations

^1^Department of Social and Preventive Medicine, University of Montreal, Montreal, QC, Canada. ^2^CESSMA (IRD-Paris-Diderot University), Université de Paris, Paris, France. ^3^Heidelberg Institute of Global Health, Medical Faculty and University Hospital, Heidelberg University, Heidelberg, Germany. ^4^CEPED (IRD-Université de Paris), Université de Paris, ERL INSERM SAGESUD, Paris, France.


Supplementary file 1. Coded Semantic Categories (English).

Supplementary file 2. Coded Semantic Categories (French).

Supplementary file 3. Interview Guide.

## Key Messages

**Implications for policy makers**

- The performance-based financing (PBF) community of practice (CoP) was one of the major catalysts of relevant policy diffusion processes on the African continent.
- Transnational policy networks such as CoPs play an important role in diffusing health systems reforms in African contexts.

**Implications for public**

Our findings highlight that despite good intentions, and while being nested in global health policy networks, influential actors coming from high-income countries (HICs) possibly drive the diffusion of health financing policies on the African continent. For those interested in policy network analysis, our study also shows that using innovative tools to make sense of large textual and relational data can help unravel the attributes and structures of these networks and their influence in policy diffusion.
