# Supplementary material for: Transnational Networks’ Contribution to Health Policy Diffusion: A Mixed Method Study of the Performance-Based Financing Community of Practice in Africa
Source: Int J Health Policy Manag. 2020 Apr 27;10(6):310–23. doi: 10.34172/ijhpm.2020.57 (PMC9056145; doi:10.34172/ijhpm.2020.57)
Supplement: Supplementary file 1 — Coded Semantic Categories (English). [file ijhpm-10-310-s001.pdf]

Supplementary file 1. Coded Semantic Categories (English)

Category title	Content (Keywords Related to Category)	N Hits
**Economics & financing**	account agenc agent allocat austerity behavi bonus capitation cash compet consumable consumer consumption contract cost demand econom efficien expenditure externalit fee financ function fund funding funds goods growth incentiv income inefficien invest liberal market maximi measur monetar money monopol neoclassic oeconom œconomi optimis optimiz paid pareto pay premium price pricing privat produc profit purchas quanti rational regulat resource revenu salar saving spending statistic subsid supply trade transfer utility value<br>*<u>*Exclusions:*</u>* "take into account" "account[ing] for" "knowledge transfer" "policy transfer" "work transfer"	**2184**
**Management**	&E administrat assessment asset autonom bank budget business capital choice client coach company debt decentrali deliver enterpr entrepr governance indicator infrastructure innovat input logistic M&E manag margin NPM operating operation outcome output planning procurement property provider-purchaser provision report reporting reports responsib result sector separat sharehold split stakeholder stock supervis supplier target technolog workflow<br>*<u>*Exclusions:*</u>* "World Bank" "German development bank" "worldbank" "the Bank" livestock (birth) deliveries "family planning" "food bank" "inputs of [someone's name] " "your input" "Federal Reserve Bank" "capitalise on your…" "Health Policy and Planning"	**1306**

**Clinical**	abortion acute antenatal Artemisinin ARTs ARVs birth body caesarean cancer cesarian chlorine chronic clinical communicable consultation contagi contracept death diabetes diagno disease eclampsia emergency episiotom equipment fetal GPs gynaeco gyneco HIV hospital hygien ICMI immuni infecti insecticide malaria maternity matron MD medical medicine midwi morbidity mortality NCD neonatal newborn nurse nutrit obstetric oxytocin paediatri pain paramed partogra patholog patient pediatri pharmac physici PNC postnatal practition pregnan prevent primary professional quality record registers registration registries registry reproduct SBA sexual sick skilled specialist STD sterilis steriliz surgeon surger surveillance symptom TB therap treatment tuberculosis uteri uterus vaccin vital vitamin weight<br>*Exclusions:* registration [to a conference/workshop/webinar]; *every proper name containing the keywords mentioned above*	**918**
**Social sciences**	anthropol beneficiar communism context [-specific] determinis diversity equality equitable equity fragil humani justice moral network poor poverty progressive redistribut respect social society socio solidarity syndical systemic union vulnerabl welfare wellbeing	**436**
**Simple buzzwords**	appropriation beneficiar capacit communit corrupt coverage data domestic empower evidence HMIS invest MDG Millennium ownership participatory partner pilot program resilien responsiven SDG strengthen sustainab techno transparen UHC universal voice vulnerb	**1126**
**PBF jargon**	AAP -based bullet CDV contract incentiv magic p4p PBF performance portal purchasing [agency] RBF scheme separat toolkit verif	**1794**
**Disagreement/conflicts**	academia academic advocate advocates bias biased clash conflict critici disagree frustrated frustrating frustration idealist idealistic inacceptable inappropriate incorrect mistake opinions opponent opposing opposition paradigm polarisation polarising polarization polarizing position proponent realistic researcher scientist unhelp	**222**

	*<u>*Exclusions:</u>* (population)'s frustrations (country or government)'s frustrations [negative sentence structure+“disagree”]	
**Agreement/ sense of community**	agree agreeing agreement belong belonging collegial colleague colleagues club cohesion common concur CoP “community of” dynamism exchange exchanging family friend friends group homogene homogeneity homogeneous homogenous join member members our participate team thank thanks us we<br>*<u>*Exclusions:</u>* “UN member” “member state/s” ”community member/s” “government member/s” “district [health] team member/s” “household member/s” “district health team/s” “government team” “family planning” “family member/s” “insurance member/s” “[first author's name] and colleague/s” US[A]	**1507**
**Africa**	*<u>*Excluded:</u>* Central Africa South Africa	**155**
**Experts**	Expert	**52**
**Normative tone**	prescri should “have to” “has to” must score rule norm normative normal	**365**
