# Supplementary material for: Transnational Networks’ Contribution to Health Policy Diffusion: A Mixed Method Study of the Performance-Based Financing Community of Practice in Africa
Source: Int J Health Policy Manag. 2020 Apr 27;10(6):310–23. doi: 10.34172/ijhpm.2020.57 (PMC9056145; doi:10.34172/ijhpm.2020.57)
Supplement: Supplementary file 2 — Coded Semantic Categories (French). [file ijhpm-10-310-s002.pdf]

Supplementary file 2. Coded semantic categories (French)

Category	Content (Keywords Related to Category)	N Hits
**Economics & financing**	account achat achet agenc agent allocat argent asymetr asymétri austerity austérité austerite behavi biens bonus capitation cash commerc compet compét comportement comptab compte concuren concurren consomm consumable consumer consumption contract contrat cost coût croissance demand depens dépense econom économi efficien expenditure externalit fee financ fonction fonds function fund funding funds goods growth incentiv incita income inefficien invest liberal libéral marché market maximi measur mesure monét monetar money monopol néoclassi neoclassic oeconom œconomi optimis optimiz paid paie paiement pareto paye premium price pricing prime privat prix produc profit purchas quanti rational rationnel rationel rationnel rationnal redevab regulat régulat rémuné rémunè remuner resource revenu salar saving spending statisti subsid subvent supply tarif trade transfer transfér utilité utility valeur value<br>*<u>*Exclusions:</u>* fonctionnaire “en fonction” fonctionnement “pris en compte” “tenir compte” “profiter de l'occasion” “j'en profite pour” demander “dans la mesure du possible” compétences “compte tenu” “tout compte fait” exprimer “pour le compte de” “tout compte fait” “au fur et à mesure” “dans la mesure où” “en mesure de”	**1764**
**Management**	actif actionnaire active approvisio assessment asset autonom bancaire banque bilan budget business capital choice cible client coach compagnie decentrali décentrali deliver dette distribution entrepr extrant fournisseur gère géré gérer gestion gouvernance indicateur infrastructure innovat input intrant logisti M&E manag marge margin monitor NPM opérationnel outcome output planning planifi prestat procurement provider-purchaser rapport rentabilité rentable reporting responsab résultat secteur separation séparation stakeholder stock supervis supplier technolog workflow	**1385**

	<u>Exclusions:</u> “Banque mondiale” “Banque africaine de développement” “banque de sang” "inputs of…" "ton/votre input" “planning familial” "sugGESTION" "sugGère" "ménaGère" "par rapport à" "rapport entre" “rapport [partagé par auteur]” "en rapport avec" "health policy and planning"	
**Clinical**	accouchem aigu antenatal anténatal Artemisin artémisin avorte birth body caesarean cancer cesarienne césarienne chirurg chlorine chronic chroniq clini communicable consultation contagi contracept CPN diabete diabète diagno disease dossier douleur ECD eclampsi éclampsi enceint episiotom épisiotom equipement équipement équiper équipés fetal fetus fétus GPs gynaeco gyneco gynéco HIV hôpit hopita hospital hygien hygièn hypertension ICMI immuni infantile infecti infirm insecticide malad malaria maternité matron medecin médecin médica medical medicament MEG morbidité mort neonat néonat nutrit obstetri obstétri oxytocin paludisme paramed paraméd partogra patholog patient PCIME pediatr pédiatr pharmac PNC postnatal practition pregnan préntal prevent prévent primaire PTME qualifié qualite qualité quality record registre reproduct seringue sexual sexue skilled soins spéciali specialist STD sterilis steriliz surgeon surger surveillance symptom TB therap traitement transmissi treatment tuberculosis urgence uteri uterus vaccin VIH vital vitamin	**1445**
**PBF jargon**	AAP ACV CDV contract contrat d'achat FBP FBR incit mécanisme p4p PBF performance portail RBF separa sépara separe sépare toolkit verif vérif	**1550**
**Buzzwords**	appropriation beneficiair bénéficiair capacit communaut corromp corrupt couverture coverage CSU data domestic domestiq durab emancip émancip empower evidence HMIS invest MDG millénaire Millennium ODD OMD partenaire participatif peren péren pilot PPP renforce resilien résilien s'appropri SDG strengthen sustainab techno transparen UHC universal universel voice voix vulnerab vulnérab SIS SNIS "données probantes"	**795**

**Social sciences**	altrui anthropol associati beneficiair bénéficiair communism context détermini determinis diversité egalit égalit equitable équitable equite équité filet fragil humani indigen justice moral pauvre progressif progressive protection redistribut resear réseau social societé socio solidaire solidarité syndica systemiq systémiq vulnérab vulnerabl welfare wellbeing<br>*__Exclusion:__* "personnalité morale"	**406**
**Disagreement/Conflict**	academi académi advocate advocates chercheur clash conflict conflit controvers critic critiq defend défend defens défens désaccord erreur excus frustr idealist idéalist idealists ideolo idéolo impos inacceptable inapproprié incorrect intolera intoléra opinions opposant oppose opposition paradigm plaidoy polaris positionnement promoteur provoca realism réalisme realist réalist recherche researcher scienti ("+anti" "+pro")	**319**
**Agreement/sense of community**	"communauté ami amis amitié bravo club cohésion collégial collégialité collègue collègues commun "communauté de »CoP d'accord d'appartenance de" dynamique dynamisme échange échanges équipe felicitations félicitations felicite félicite féliciter fraternel groupe homogène membre membres merci nos nous partagez participez rejoignez rejoins (+ "je soutiens" "je souscris")<br>*__Exclusions:__* "intérêt commun" "fond commun"	**1285**
**Africa**	Afrique africain<br>*__Exclusions:__* "Afrique du Sud" "Banque africaine de développement"	**102**
**Experts**	expert	**71**
**Normative tone**	devraient devrais devrait dois doit doivent normal normatif normative norme normes règle règles respectée respectées respecter score	**454**
