# Supplementary material for: Transnational Networks’ Contribution to Health Policy Diffusion: A Mixed Method Study of the Performance-Based Financing Community of Practice in Africa
Source: Int J Health Policy Manag. 2020 Apr 27;10(6):310–23. doi: 10.34172/ijhpm.2020.57 (PMC9056145; doi:10.34172/ijhpm.2020.57)
Supplement: Supplementary file 3 — Interview Guide. [file ijhpm-10-310-s003.pdf]

**Supplementary file 3.** Interview guide

**Focus of the interview: Assessing the diffusion of performance-based financing on the African continent**

- *This interview is designed to be carried out as a friendly, open conversation about the diffusion of a global health policy in Africa: performance-based financing (PBF)*
- *The purpose is to explore how ideas (eg, economic theories), knowledge (eg, past and present research evidence, grey literature), and networks contributed to the development of the policy in low- and middle-income countries.*
- *The aim of the interview is to understand and accurately describe ideas and knowledge mobilisation in the development of the PBF policy.*
- *Interviews are audio recorded, unless notified otherwise by the interviewee.*
- *The structure below is a guide. The conversation might not always be this linear, however all questions ought to be addressed before the interview concludes. Interviews should take up to 1.5 hour.*
- *The prompts in italics can be used as supplementary questions but should not replace the main question they are attached to. On some instances, an interviewee might touch upon important themes: these may be re-employed by the interviewer using the interviewee's own words, in an attempt to make the interviewer talk about the mentioned theme in relation to the PBF policy process specifically.*

**The Interview**

| | | | | | |
|---|---|---|---|---|---|
| **Archival Number** | | | **Name of Interviewee** | | |
| **Position & Affiliation of Interviewee** | | | | | |
| **Name of Interviewer** | | | | | |
| **Mode of Interview** | | | Face to face ◯ Phone ◯ | | |
| **Audio recorded** | | | Yes ◯ No ◯ | | |
| **Date** | | | **DD** | **MM** | **YYYY** |
| **Start Time** | | | **End Time** | | |

I am XXX, a PhD student at XXX and XXX. The aim of this research project is to learn about the processes that led to PBF diffusion in African countries. This study is part of a larger research evaluation program on "Results-based financing for equitable access to maternal and child health care in Mali and Burkina Faso." This program is run by a consortium of Mali- and Burkina Faso-based research NGOs

and researchers from the University of Montreal[1]. I have asked to interview you, because you are a key person with knowledge and insight regarding the emergence of PBF as a global health policy and I am interested in learning your opinions and personal experience regarding the PBF policy.

I have reviewed the procedures for the interview during the consent process. Do you have any further questions before we begin?

**A. Section on Representation Systems, Motivations and Resources**

| Question | Prompts | Comments |
|---|---|---|
| **1. Could you tell me a little bit about your background?** | *How did you come to your current position? What was your personal trajectory?* | |
| **2. How long have you been working in this institution?** | *What is the purpose of the PBF policy? What policy issues does it address?* | |
| **3. How do you feel about your job?** | *What would be the most fulfilling or exciting, or the most challenging or frustrating in your job?* | |
| **4. How did you first hear of performance-based financing (PBF)?** | | |

| | | |
|---|---|---|
| **5. For you, what does this policy represent in African countries?** | | |
| **6. According to you, what helped the most in shaping the development of this policy?** | *What impact did this have? How much help were they?*<br>Facilitators might relate to:<br>• Individual skills/knowledge<br>• Positive evaluations<br>• Political factors<br>• Policy/program topic factors | |
| **7. Could you tell me a little bit about the ideas and values that are behind the development of PBF in Africa?** | *What is the purpose of the PBF policy? What policy issues does it address?* | |
| **8. According to you, what are the possible reasons for choosing to implement this policy in many African countries?** | | |
| **9. What do you think about the effect(s) of policy?** | *Do you believe that this policy works well in LMICs? Why or why not?* | |
| **<u>B. SECTION ON STRATEGIES</u>** | | |

[1] This project is part of the Innovating for Maternal and Child Health in Africa (IMCHA) Initiative, a seven year, $CA36 million multi-donor partnership funded by Global Affairs Canada, the Canadian Institutes of Health Research, and Canada's International Development Research Centre

| | |
|---|---|
| **10. What types of documents are used about PBF and why do you think they are relevant resources?** | • *Primary research studies; such as impact evaluations published in academic journals*<br>• *Secondary research articles (reviews) such as systematic reviews or research summaries*<br>• *Govt. reports or other unpublished (grey) literature, including evaluations of policies or programs from other organisations* |
| **11. How is the relevance of the research or of the methods used in impact evaluation determined?** | *Was an assessment made of whether the research was:*<br>• *Consistent with previous research?*<br>• *Compatible with organisational values/knowledge?*<br>• *Actionable/feasible?* |
| **12. Would you consider that evidence (any type of knowledge resources) informed the development of the PBF policy in any way? How did they?** | *What is your assessment of the importance of the contribution of knowledge resources to the development of the PBF policy overall?* |
| **13. For instance, can you name a specific knowledge resource on PBF used in your organisation?** | |
| **14. Would you say that this knowledge was used to persuade others to a point of view or course of action?** | *Can you describe how research was used?*<br>*Which stakeholders were they trying to persuade/inform/ justify the decision to?* |
| **15. Do you have concerns about how these resources were used in the development of PBF in countries?** | *Were there any consequences of the use or non-use of these resources?*<br>*Were there concerns about the use of generalised research for instance?* |
| ***"Having explored the ways knowledge was used, I would now like to talk about the other elements that may have played a role in policy-making"*** | |
| **16. Did you play a specific role in the development and/or diffusion of this policy on the African continent? If yes, what was this role about?** | NB: Understanding the role of the interviewee is very important to the validity of the interview. Was their role central, marginal, advisory? Did they produce/contribute to/direct some documents on PBF? |
| **17. With what type of people do you work with on PBF?** | |
| **18. Could you tell me about the consultation process with experts, reference groups or researchers to inform this policy?** | |
| **19. As you may know, the *Health Results Innovation Trust Fund* facility funds a range of activities for governments' representatives and local experts to familiarise with PBF. Do you participate in these activities? Why or why not?** | *Did you already participate in a PBF training and/or PBF-related country visit?*<br>*To at extent do these activities contribute to policy-making in African countries according to you? Could you give some examples?* |
| **20. If yes, how many times have your participated in these activities?** | |
| **21. Could you please describe for me how these activities take place and what their objectives may be? You may give a particular example if you wish.** | |
| **22. In your opinion, how do training or country visits help convince national decision makers?** | |

| | |
|---|---|
| **23. Who do you approach when you need expertise on PBF?** | |
| **24. Why do you choose to approach these people in particular?** | |
| **25. Who do you approach when you need advice to help convince national governments of the value of PBF?** | |
| **26. Why do you choose to approach these people in particular?** | |
| **27. What type of advice do these resource people give you?** | |
| **28. In your opinion, how does advice from these resource people help convince national decision makers?** | |
| **29. In your opinion, how do these resource people influence the diffusion of the PBF policy idea on the African continent?** | |
| **30. Is PBF in the national policy of your home country?** | |
| **31. Have you contributed to organise one of these activities? If you have, how are government representatives selected to participate in these activities?** | |
| **32. Why do you think these activities are popular among African participants?** | |
| **33. Do you participate to the discussions on PBF, such as through the Community of Practice or web conferences on PBF?** | *If yes, what do you think about it?*<br>*If no, why not?*<br>*To what extent these discussions inform countries decision makers according to you?* |
| **34. In your opinion, what has been the response by African governments to PBF? Why?** | |
| **35. Who else do you think it is important that we speak to (if more than one person nominated, ask interviewee to rank in order of relevance/importance)?** | |
| **36. Are there any resources or documents you would recommend we consider?** | |
| **37. Is there anything you think has been missed or that you think is important for us to be aware of?** | |
